# Ozæna

**Published:** 1907-07-20

**Authors:** 


### Laryngology and Rhinology.

### OZÆNA.

The term "ozæna," derived from ὄζη, a stench, implies simply a stinking condition of the nose, which may be produced by various causes, such as syphilitic ulceration, necrosis, foreign body, or neglected sinus-suppuration; the commonest cause, however, is a condition known as "atrophic rhinitis," or true ozæna, which presents certain definite characters, and whose nature and causation have been, and still are, the subject of much controversy.

The principal characteristics of this affection are as follows. Females are affected more than males in the proportion of about three to one; the disease is generally stated, on the authority of Morell Mackenzie, to begin about the age of fifteen or seventeen, but it is certainly often found before that period. A peculiar physiognomy is described; the face is broad and the skull brachycephalic; the bridge of the nose is wide and flat, and the nostrils, too, are broad, and so directed forwards as to be unusually conspicuous. The appearance differs from the "saddle-back" deformity of congenital syphilis, in that the bridge, though flat, is straight and not concave in profile. These characters are, however, found in rather less than half the cases, and all persons with this aspect do not suffer from ozæna. The influence of heredity is often marked, the disease attacking those members of a family with the facies mentioned above.

The inferior turbinals are small and the nose is very roomy, so that the naso-pharynx can often be seen; the middle turbinals are sometimes enlarged and polypoid. The discharge consists, not of true pus, but of mucus mixed with shed epithelial cells and débris; this collects and dries into large greenish-black crusts, and gives rise to a peculiar sweetish and horribly offensive odour. There may be some superficial erosion, but never deep ulceration, necrosis, or septal perforation. Similar crusting may extend to the pharynx, larynx, and even the trachea. The sense of smell is usually lost, so that the patient is not herself conscious of the fœtor. Over the inferior turbinals the columnar ciliated cells are replaced by squamous epithelium; the submucous glands are degenerated, the venous sinuses have disappeared, and the entire mucous membrane is thinner and more fibrous than normal.

Many hypotheses have been advanced to explain the causation of this disease, but it cannot be said that any has been definitely proved as yet. Grünwald considers that it is due to disease of the accessory sinuses. Sinus disease may perhaps cause atrophic rhinitis in a minority of cases in a manner to be explained later, and it is sometimes found as a complication, the sinuses being infected from the nose. It has also been said that ozæna is the final stage of rhinitis sicca or of hypertrophic rhinitis, that it is caused by the bacilli of tuberculosis, syphilis, diphtheria, or other specific disease, or that it is a kind of tropho-neurosis; but none of these conjectures fits the facts. Hofmann's theory that it is due to a congenital predisposition evidenced by a brachycephalic skull and short, wide nose, is insufficient; the wide nose is doubtless a predisposing condition, but this type of face is present in barely half the cases.

To regard ozæna as a sequel of purulent rhinitis in childhood—an idea put forward by Bosworth and recently supported by Lack—seems in closer agreement with the clinical facts, and so to afford a rational basis for treatment. Suppurative rhinitis is accompanied by desquamation of the epithelium, and, if long continued, results in replacement of the ciliated by squamous cells; the chief agent for the removal of the secretion is thus lost, and retention is materially assisted by any undue width of the nasal fossæ, which diminishes the force of the current of air and tends to dry the secretion. Thus crusts are formed and decompose, and non-development of the turbinals (which results from long-continued inflammation) causes a further widening of the nasal passage. The ciliated epithelium is lost for ever, and therefore the disease is not completely curable.

Treatment depends on our hypothesis of the nature of the affection. Thus diphtheria antitoxin has been employed on the assumption that the prime cause is the Klebs-Loeffler bacillus, and stimulants have been applied under the idea that atrophy of the mucosa is the essential lesion; but these have not proved successful, and the following method, based on the theory last discussed, gives better results. The nose must be kept clean from crusts by syringing; there is no specific virtue in any drug, but the lotion should be alkaline and mildly antiseptic, and plenty must be used. The surgeon should convince himself that this is properly done at the beginning of the treatment, and himself remove adherent crusts. Next crusting must be prevented, and the only certain way to do this is to exclude air; this may be done by plugging the anterior nares with wool, but it is better to lightly pack the inferior meatus with gauze moistened with oil. The gauze is worn continuously, removed morning and evening, and, after syringing, a fresh strip inserted. The patient is taught to do this, and in this way the fœtor is immediately abolished. After some weeks or months the packing is omitted and, if the nose remain free from fœtid crusts, it is left off entirely, but syringing and spraying with oil must be continued indefinitely. The secretion remains liquid when air is excluded, and it is seen to be mucoid, and not purulent. After the nose has been packed for a few days, the surgeon should, on removing the gauze, make a careful search for pus; if any be seen, disease of the accessory sinuses should be suspected. The pus must be localised, followed to its source, and the affected cavity explored. General treatment is important, and includes tonics, cod-liver oil, and change of air, especially to the seaside.

